# Mandarin and English Event Cognitive Alignment From Corpus-Based Semantic Fusion Model Perspective

**DOI:** 10.3389/fpsyg.2022.872145

**Published:** 2022-05-06

**Authors:** Xiangling Li

**Affiliations:** Institute of Foreign Linguistics and Applied Linguistics, Henan University, Kaifeng, China

**Keywords:** semantic role, semantic fusion, event structure, semantic profile, event alignment

## Abstract

The study explores the fusion of semantic roles and the different semantic fusion types, aiming at establishing a semantic fusion model to explain the cognitive alignment of events in Chinese and English simple sentence constructions containing two verbs. In total, 20,280 simple sentence constructions containing two verbs are collected from Chinese literary works, Peking University Chinese Corpus, and English classic literary works. The semantic fusion in the collected simple sentence constructions containing two verbs is classified into five major semantic fusion categories, which appear with different occurrence frequencies in the two languages. The semantic fusion model of event alignment is comprehensively supported by linguistic research in Chinese and English. From a cognitive linguistic perspective, it is found that the double semantic profiles of the same syntactic element N (noun) make N psychologically activated twice and enable it to enter two processes profiled by the two verbs as a participant. The two processes are combined into one event, which designates a cognitive occurrence of any degree of complexity. N’s entry into the two subevents is realized by its double semantic profiles that enable it to fuse two semantic roles into one syntactic element and explain the relationship between N’s double syntactic identities and double semantic roles. The semantic fusion model was used to explore event alignment in simple sentence constructions containing two verbs, and it was discovered that the fusion of two semantic roles is universal in languages and is a common psychological and cognitive behavior deeply rooted in the mental conceptualization of language users. The empirical discussion of simple sentence constructions containing two verbs proves that semantic fusion as an important psychological passage in event alignment has solid psychological reality and verifies the applicability of the semantic fusion model in the explanation of event alignment.

## Introduction

Semantic roles have a long-standing presence in theories of philosophy, cognitive science, and linguistics. The semantic roles such as agent, patient, goal, and instrument are cross-culturally universal (Fillmore, 1968) and are regarded as part of innate core language knowledge (Carey, 2011; Strickland, 2017). For a long time, semantic roles are routinely involved in the studies of language production, language acquisition, the interface between syntax and semantics, and cognitive science. The verb “eat” encodes a semantic relation between someone who eats and something that gets eaten, and the participants involved in this relation are given the role labels “agent” and “patient,” respectively (Rissman and Majid, 2019). Semantic roles are very common in that they are fundamental to how people represent the world and how these representations are expressed in language. As a common semantic relationship, semantic fusion refers to the merging of two or more semantic roles and is an important means of event cognitive alignment in Chinese and English. Semantic fusion makes for the succinctness of language expressions; different actions within a simple sentence construction containing two verbs are integrated into one complete event through the fusion of two semantic roles. In the sentence “The boss made Tom do the work all day,” the actions “make” and “do” are integrated into an event by way of the shared participant “Tom,” with “Tom” being the patient of the action “make” and the agent of the action “do.” The fusion of two semantic roles is common in English simple sentence constructions containing two verbs, such as resultative constructions and caused-motion constructions. Similarly, semantic fusion is very pervasive in Chinese simple sentence constructions containing two verbs, such as Chinese pivotal constructions, Chinese constructions with serial verbs, and verb-complement constructions. The semantic fusion in a simple sentence construction containing two verbs is the research focus of this study.

## Literature Review

The research of events has been a classic topic of concern in the field of philosophy. Events are divided into actuality and movement, which is regarded as the earliest study of events. Vendler (1957) further divides events into state, activity, achievement, and accomplishment. Davidson (1967) puts forward the concepts of event argument and event individualization and establishes the ontological position of events. In the field of psychology, events are also extensively studied, especially the psychological authenticity of events (Shipley and Zacks, 2008), the causative relationship between an event and the speaker, a causal relationship between the event and the state, the psychological relationship with the action event, and the simultaneous relationship between the state and the event (Kistler, 2006; Chen, 2021). Event-related brain potential (ERP) studies provided evidence in support of parallel lexical access during bilingual language production (Wu and Thierry, 2011).

In the field of linguistics, the study of events is also a key topic for half a century (Jackendoff, 1976, 1990; Talmy, 1985; Pinker, 1989; Rappaport, 2008; Viswanatha et al., 2018; Li F. Y., 2019). Talmy (1985) puts forward a mobile event conceptual framework and the theory of macro-events, defining events as macro-event = motion-event + co-event, with secondary events indicating the way the main event moves or the reason for the movement of the main event (Talmy, 2000a). Pinker (1989) examines the relationship between syntax and semantics through the decomposition of predicate meaning and points out that the meaning of verbs is decomposed into major events and minor events, which are represented by a tree diagram method. In essence, although Pinker and Talmy’s macro-event theories are expressed in different ways, they are somewhat similar. Jackendoff (1976) emphasizes that any event such as motion and spatial location is based on the basic predicate verbs and the interrelationship between causative verbs. Rappaport (2008) proposes semantic decomposition and believes that the internal semantics of verbs include root meaning and structural meaning. In short, the syntactic representation of semantic roles in an event and the analysis of the semantic structure of an event gradually arouse great interest in the field of linguistics.

Within the framework of Chinese traditional grammar, Zhang (1999,^2001^), by exploring Chinese pivotal constructions in the oracle bone script and Chinese sentences with serial verbs in the inscriptions of the Western Zhou Dynasty, point out the double syntactic identity of the same linguistic form in some special sentences, which gradually becomes the focus of debate among scholars. The psychological processing of squeezing two semantic components into one syntactic form is a common sentence-making method in Chinese (Lv, 1979).

The syntactic representation of simple sentence constructions containing two verbs also attracts scholars’ observation from the perspective of structural linguistics. Under the influence of behaviorism, which holds that meaning is the situation expressed by a linguistic form and the response aroused in the listeners (Bloomfield, 2002), the structural research attaches great importance to linguistic form and proposes immediate constituent (IC) analysis to analyze the double syntactic identity of one syntactic element and puts forward dividing-one-word–into-two hypothesis to explain why the same syntactic element can merge or fuse two semantic roles in Chinese (Xing, 1986; Wu and Liang, 1992).

Logical analysis of the event structure in simple sentence constructions containing two verbs is carried out by scholars within the theoretical framework of transformational and generative linguistics. In accordance with the Thematic Criterion of the Governing & Binding theory, a theme can only be assigned one and at most one thematic role, and each thematic role can only be assigned to one theme (Huang, 1982; Boeckx and Horstein, 2004; Chomsky, 2010). The previous studies from the perspective of generative grammar believe that the syntactic element with two semantic roles violates the Thematic Criterion at the syntactic level, cannot have two syntactic identities at the same time, and puts forward an empty category (abbreviated to e) to explain the fusion of two semantic roles within one linguistic element. In the deep structure of a simple sentence construction including two verbs, there is an empty category behind the syntactic element that plays two semantic roles. In the English sentence “Tom persuaded Janie (e_*i*_) to go to a picnic,” there is an empty category e_*i*_ behind Janie, and the empty category e_*i*_ also refers to Janie. The proposal of empty category gives a satisfactory answer to the fusion of two semantic roles in a linguistic form and probes deeper into the logical structure, which helps to make clear the semantic structure of the event. The invisibility of the empty category at the syntactic level and its appearance at the semantic level touch upon the psychological representation of event structure in a simple sentence construction containing two verbs (Xing, 2004; Feng and Feng, 2018). However, why is there an empty category hidden behind the syntactic element, why does it turn up in semantic structure, and why it is shaded in the syntactic structure are still some doubts that need further explanation.

The studies on semantic fusion from the perspective of cognitive linguistics and cognitive psychology gradually arouse more attention. Goldberg (1995) pointed out that role merging occurs in reflexive constructions, with one participant role merging with another. The merged participant roles are squeezed with a single argument role and linked with a single grammatical function. Two actions in a sentence are integrated by merging two participant roles into one single argument role to form a composite event that is linked with a single grammatical function. The event participant categories are not as self-evident as categories provided by nouns and verbs (Rissman and Majid, 2019), and a variety of event-specific knowledge is activated during sentence comprehension (Bicknell et al., 2010; Metusalema et al., 2012). Talmy (2000b) discovered that a simple sentence representing an event is universal; it is not language-specific. The typical characteristics isomorphism between the event cognition and linguistic representation and the isomorphism between semantic fusion and syntactic fusion of an event were examined (Davidson, 1967; Parsons, 1990; Croft, 1991; Talmy, 2000b; Givón, 2001; Imbert, 2012; Li, 2020). The previous studies of events from a cognitive perspective are roughly divided into two categories. The first category takes verbs as the core, which focuses on the event structure and the realization of arguments (Jackendoff, 1990; Levin, 1993; Croft, 2012). The second category focuses on the difference in the linguistic representation of event components. Event integration through the fusion of semantic roles arouses scholars’ great interest (Talmy, 1991; Fauconnier and Turner, 1996; Givón, 2001).

From the perspective of cognitive psychology, the natural language sentence matching method is proposed to combine high-level and low-level semantic information, using a heuristic fusion function to merge low-level semantic information with high-level semantic information to get the final semantic representation (Jiang et al., 2021). With regard to the mapping between syntactic relations and semantic cases, Van (2005); Gruber (1965), and Fillmore (1968) discovered that there exists a correspondence between semantic roles and syntactic locations. Jackendoff (1983) advocated that the argument structure should be described using complex and clear semantic structure, which is mapped to the syntactic structure. Croft (2012) analyzes the direct mappings between specific event structures and syntactic positions (e.g., subject and object) (Rissman and Majid, 2019). Fuzzy semantic overlapping allows a member to belong to more than one community (Sato et al., 2020). Similarly, in language, semantic fusion helps a participant to enter two actions and link them into a composite event (Langacker, 2012). The research on simple sentence constructions with one subject and two verbs in Chinese and English discovers one interesting fact that the verbs in them share at least one argument role that plays two participant roles. Squeezing the two roles into one word is crucial in the psychological alignment of an event. Semantic fusion seems to be a basic way for people to copy and combine different scenes into a complete human scene in the objective world (Li, 2015; Li X. L., 2019; Liu, 2017; Wen and Yin, 2018; Zhang and Pan, 2019). The cognitive mechanism that enables bilinguals to keep their languages functionally operating has not yet been elucidated (Wu and Thierry, 2017). So, in order to reveal the psychological and cognitive mechanism of this kind of language phenomenon, an in-depth research is needed.

In summary, previous studies on events in Chinese and English are done from different linguistic approaches. The existence of double syntactic identity is the biggest discovery in the previous studies from the perspective of traditional grammar and has aroused heated discussion, but the studies from the traditional grammar cannot explain the reason why the same linguistic form possesses two syntactic identities. The studies within the framework of structural linguistics put forward the dividing-one-word–into-two hypothesis to expound the double identity of the same linguistic form but still cannot explain why one word can be divided into two words at the syntactic level. The studies from the approach of generative linguistics put forward an empty category to offer a very convincing explanation to the double syntactic identity of the same linguistic form with the help of thematic role theory, pushing forward the studies of the events grounded in language, but at the same time leave one doubt why there exists an empty category behind one syntactic form. The cognitive studies of the event in simple sentence constructions attracted more and more attention, and many scholars try to explain the event structure and event integration from a cognitive perspective. But why one syntactic element can play two semantic roles needs to be probed further in order to reveal the cognitive mechanism of event integration in a simple sentence construction containing two verbs.

## Theoretical Guidance

Research is carried out with the guidance of Gestalt psychology and cognitive linguistics. Gestalt psychology emphasizes the integrity of experience and behavior and studies objects as a whole, which is not equal to the sum of the parts. The whole precedes the parts and determines the nature and meaning of each part. According to the principle of good Gestalt, the parts that belong to each other are easy to combine into a whole; on the contrary, the parts that do not belong to each other are easy to be isolated (Blackburn, 1940). Simplicity is one of the perception principles. When people perceive things, they often grasp the overall objective object through specific characteristics of certain parts and tend to summarize complex things into concise shapes by combining inherent experience and cognition (Chen, 2021). According to the shortest distance principle or proximity factor, some parts that are close to each other are easy to form a whole (Koffka, 1935).

Cognitive linguistics regards “Language is an integral part of human cognition” (Langacker, 1987:12). Composition is the starting point of cognitive linguistics. Different from the traditional valence theory, the cognitive valence theory believes that the valence relationship refers to the composition relationship between two or more linguistic units (Langacker, 1987). The valence relationship between linguistic units is established through the corresponding relationship between the semantic profiles of two semantic components. In accordance with the valence theory of cognitive grammar, a noun profiles a thing, a verb profiles a process, and an adjective or adverb profiles an atemporal relation. Based on this valence theory, Niu (2008) proposes a cognitive analysis model, which provides access to the combination of semantic components within a composite linguistic unit. The semantics of a grammatical structure not only contains objective and real conceptual information but also entails language users’ perception, cognition, construal, and reasoning of objective reality. Talmy (2000a) believes that sentences can represent a series of cross-event relations, including time, cause and effect, concessions, and attachments. The typical feature of event fusion is that people’s cognition and linguistic representation of events appear in isomorphism (Talmy, 2000b; Givón, 2001). The fusion of cause and effect is an important feature of human cognition. People have widely accepted that the cause event and the effect event are conceptualized as macro-event (Michotte, 1946; Talmy, 1991, 2000b; Wolff, 2003). The sub-events containing causal relationships can be merged into a single macro-event and represented by a single sentence. In this study, Gestalt psychology and cognitive linguistics are used to investigate the cognitive alignment of events in English and Chinese simple sentence constructions containing two verbs by way of semantic fusion.

## Materials and Methods

In the study, a large number of data are collected in order to analyze and classify the different categories of semantic fusion types. A total of 20,280 Chinese and English simple sentence constructions containing two verbs are collected from the Chinese classical literary works, Peking University Chinese Corpus, and English classic literary works. All the collected simple sentence constructions containing two verbs are classified according to the semantic fusion types, and the occurrence frequency of different semantic fusion types is counted. Based on the analysis of the semantic roles and the fusion types, a quantitative method is used to classify semantic fusion into different categories. Based on the qualitative analysis of semantic fusion realization and the noun’s function in the integration of the two actions, an event semantic fusion model is established. The qualitative method is used in the explanation of how the two actions (sub-events) are integrated to form a composite event from a psychological and cognitive approach. Qualitative analysis is also adopted to expound what is the universally applicable cognitive mechanism for the combination of sub-events in a construction. The corpus-based quantitative method and the introspective qualitative method make the semantic fusion model possible and reasonable. Finally, an empirical discussion is done by carrying out a test. A total of 48 participants who are linguistic postgraduates took part in the test. The test is composed of 20 simple sentence constructions in Chinese and English and five types of semantic fusion. In total, 15 sentences are in the form of SV_1_NV_2_, two sentences are in the form of SV_1_V_2_N, and three sentences are in the form of SV_1_NA(Adj.). The 20 sentences are put together in order to check whether the participants can accurately differentiate the three forms of constructions. In the test, the participants are asked to finish the following three tasks: (1) to recognize the N that plays two semantic roles, (2) to make a judgment whether the constructions in the test are SV_1_NV_2_ constructions, and (3) to determine whether the two verbs V_1_ and V_2_ are combined into a complete event through the syntactic element N. The test is used to testify N’s function in the event integration of the two actions V_1_ and V_2_ and to prove that semantic fusion model is feasible and applicable in explaining the event integration.

## Linguistic Representation of Semantic Fusion in Simple Sentence Constructions Containing Two Verbs

In this section, the linguistic representation of simple sentence constructions including two verbs is discussed in detail. Simple sentence constructions containing two verbs have two typical syntactic features: (i) There are four indispensable elements in the construction: two nouns, one of which is the subject, and two verbs, usually in the form of SV_1_NV_2_ or SV_1_V_2_N. (ii) N usually appears before, between, or behind the two verbs.


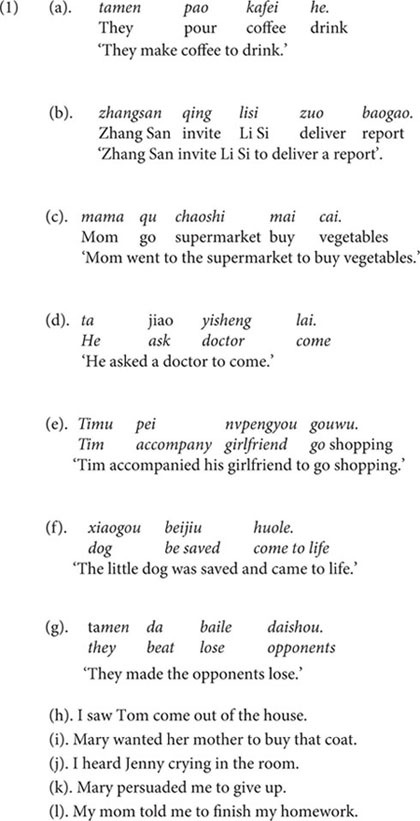


In (1), a–g are the simple sentence constructions with two verbs in Chinese. (1) h–l are simple sentence constructions with two verbs in English. In these constructions, the noun (N) is very crucial, and it is usually between the two verbs as it is seen in (1) a, b, d, e, i, and k. In (1) a, *kafei* “coffee” is put between the two verbs *pao* “make” and *he* “drink,” and in (1) b, *lisi* “Li Si” is between the verbs *qing* “invite” and *zuobaogao* “make a report.” In some of these constructions, the noun (N) is behind the two verbs. In (1) g, the noun *daishou* “opponent” is put behind the verbs *da* “beat” and *bai* “lose.” The complexity of (1) e needs a detailed analysis. In (1) e, although there are two verbs *pei* “accompany” and *gouwu* “go shopping,” there are two nouns *timu* “Tim” and *nvpengyou* “his girlfriend,” and both of the two nouns have a syntactic relationship with the two verbs.

## Classification of Semantic Fusion in Simple Sentence Constructions Containing Two Verbs

The semantic role generally refers to the role of the participant in the event or activity described by the predicate. In the study of syntax and semantics, this participant role has been given many different names, such as deep case (Fillmore, 1968), thematic roles (Gruber, 1965; Jackendoff, 1972; Dowty, 1986; Carlson, 1998), participant roles (Allan, 1986), semantic roles (Givón, 1990), and argument roles (Goldberg, 1995). The deep structure of a sentence includes a predicate and one or more noun phrases, and each noun phrase establishes a specific case relationship with the predicate (Fillmore, 1968). Agent, experiencer, patient, theme (undergoer), fractive, and locative are six basic semantic roles.

Semantic fusion is very complex and pervasive, appearing in different combinations of semantic roles. Based on the observation and analysis of 20,820 simple sentence constructions (15,715 in Chinese and 5,105 in English) collected from the corpus and sources mentioned above, it is discovered that there are mainly five semantic fusion categories (refer to Table 1): agent-agent fusion, agent-patient fusion, agent-experiencer fusion, patient-patient fusion, and patient-experiencer fusion.

**TABLE 1:** Categories of semantic fusion in Chinese and English.

Corpus Type	Chinese classic literary works		Peking University Chinese Corpus		English classic literary works		Total	
	Quantity amount	percentage	quantity	percentage	quantity	percentage	quantity	percentage
Type I agent /agent fusion	2980	30.21%	1880	32.14%	1650	32.32%	6510	31.27%
Type II agent /patient fusion	2660	26.96%	1660	28.38%	1550	30.36%	5870	28.19%
Type III agent /experiencer fusion	1805	18.30%	980	16.75%	860	16.85%	3645	17.51%
Type IV patient /patient fusion	1255	12.72%	670	11.45%	520	10.19%	2445	11.74%
Type V patient /experiencer fusion	1165	11.81%	660	11.28%	525	10.28%	2350	11.29%
Total	9865	100%	5850	100%	5105	100%	20820	100%

Type I agent/agent fusion is the type of semantic fusion with the highest occurrence frequency and accounts for 31.27% of the data collected. *mama* “mom” is the agent of the action *quchaoshi* “go to supermarket” and the agent of the action *maicai* “buy vegetables” in (1) c. In (1) e, the two actions *pei* “accompany” and *gouwu* “go shopping” share the same agent *xiaowang* “Xiaowang.”

Type II agent/patient fusion occurs with relatively high occurrence frequency, *occupying* 28.19% of the data collected. This type of fusion is discovered in (1) b, d, and f. In (1) b, lisi “Li Si” is the patient of the action *qing* “invite” and the agent of the action *zuobaogao* “deliver a report”; in (1) d, *yisheng* “doctor” is the patient of the action expressed by the verb *jiao* “ask” and the agent of the action expressed by the verb *lai* “come”; in (1) f, *xiaogou* “little dog” is the patient of the action *beijiu* “saved” and the agent of the action *huo* “come to life.”

The frequency of type III agent/experiencer fusion is slightly low and takes up 17.51% of the data collected. In (1) e, *nvpengyou* “his girlfriend” is the experiencer of the action expressed by the verb *pei* “accompany” and the agent of the action indicated by the verb *gouwu* “go shopping”; in (1) h, “Tom” is the experiencer of the action expressed by the verb “see” and the agent of the action performed by the verb “come”; in (1) j, “Jenny” is the experiencer of the action performed by the verb “hear” and the agent of the action expressed by “cry.”

Similar to the first three types of semantic fusion type discussed earlier, the fusion type of patient/patient and patient/experiencer is also pervasive in language, but when compared to the first three fusion types, the occurrence frequency of these two types is slightly lower with 11.29 and 11.74%, respectively. Patient/patient fusion appears in (1) a, where *kafei* “coffee” is the patient of the action expressed by the verb *pao* “pour” and the patient of the action expressed by the verb *he* “drink.” Patient/experiencer fusion is discovered in (1) g, where *daishou* “opponent” is the patient of the action *da* “beat” and the experiencer of the action *bai* “lose.”

## Semantic Representation of the Event in Simple Sentence Constructions Containing Two Verbs

The semantic representation of these constructions involves semantic decomposition. By decomposing the meaning of a word into various aspects (components, means, participants, location, etc.), what is latent in the meaning of a word is made apparent. In simple sentence constructions containing two verbs, the understanding of the meaning of V_1_ and V_2_ is closely related to their arguments. The semantics of simple sentence constructions consists of two basic parts: (1) the representation of semantic components and (2) the representation of the event logical structure. In simple sentence constructions containing two verbs, the predicate is represented by an activity logical structure that has three arguments. In these constructions, the predicate V_1_ takes three arguments, with V_2_ being one of them. Semantic roles are the roles that arguments of a predicate take. Consider the sentence “Joe squeezed the rubber ball inside the jar,” “squeezed” is the predicate. “Joe, rubber ball and jar” gets the semantic roles of squeezer (agent), squeezee (patient), and location. This motion event is described by a verb (squeeze), a proper noun (Joe), a noun phrase (the rubber ball), and a preposition phrase (inside the jar). A neo-Davidsonian event representation of this motion event is as follows: ∃ e,x,y Squeezing(e)∧Squeezer(e, Joe)∧Squeezed Thing(e,y)∧Rubber Ball(y).

Similarly, in simple sentence constructions containing two verbs, the semantic roles express the roles that arguments of V_1_ and V_2_ take. The semantic representation of the event structure is as follows: ∃e, S, N, (X) V_1_-ing(e1)∧V1-er (e1, S)∧V_1_-ed Thing(e1, N)∧V_2_-ing(e2)∧V_2_-er (e2, N)∧V2-ed Thing(e2, x).

This formula encodes an event, and the participants are S, N, and X. S and N are two indispensable participants, and X is not the necessary participant. S and N are the participants of sub-event 1 expressed by V_1_. N and X are participants of the sub-event 2 expressed by V_2_. The event in (1) k is as follows: ∃e, Mary, me, x Persuading (e1)∧Persuader (e1, Mary)∧Persuaded Thing (e1, me)∧Giving up (e2)∧give-up-er (e2, me)∧given up Thing (e2, x).

## Semantic Fusion Model of Event Alignment

**FIGURE 1:**
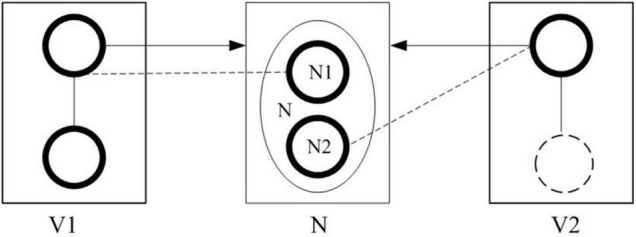
Semantic fusion model of event cognitive alignment.

Through the analysis of semantic fusion types, it is discovered that the semantic components in a construction express a complete meaning and are regarded as a whole. Here the cognitive alignment of the semantic components in a sentence is expounded with the guidance of cognitive linguistics. According to the valence theory of cognitive grammar, the three semantic components are aligned into a complete event by the way of semantic profiling, and the alignment process is shown in Figure 1.

In Figure 1, N is an autonomous element; V_1_ and V_2_ are dependent elements. N profiles an entity with one substructure elevated to a special level of prominence. The bold circle in the boxes V_1_, N, and V_2_ stands for the profiled substructure. The box stands for the base, which refers to the basic cognitive domain used to perceive the profiled substructure. The left and right boxes stand for the process or relation profiled by V_1_ and V_2_. The dotted circle in the right box stands for the possible existence of profiled substructures of V_2_. The dotted line stands for the correspondence between the profiled structure of the dependent element and the profiled structure of the autonomous element. The arrow stands for the elaboration relation in which one element provides an elaboration site that is elaborated by the profiled structure of another element in construction.

In simple sentence constructions containing two verbs, the two verbs (V_1_ and V_2_) are dependent elements that each profiles a process that includes one or two participants. The noun (N) semantically profiles an entity. In the composition between V_1_ and N, V_1_ provides an elaboration site, and one semantic profile of N (N_1_) is psychologically activated to elaborate the site and helps N get its entry into the process profiled by V_1_. N becomes one of the participants of the process. In the composition between N and V_2_, V_2_ profiles a schematic trajector, and another semantic substructure of N (N_2_) is mentally activated and elaborates the schematic trajector profiled by V_2_. Distinct and related predications are obtained by imposing alternate profiles on a given base (Langacker, 1987). Alternate profiles of N are psychologically activated and enable it to enter two processes as a participant, and the two processes are combined into one event, which designates a cognitive occurrence of any degree of complexity. N’s entry into two processes is realized by its double semantic profiles, which explains why the two semantic roles of N are fused into one participant. N becomes a psychological passage in the combination of two processes with the help of its double semantic profiles.

## Discussion of Event Alignment Through Semantic Fusion Model in Simple Sentence Constructions Containing Two Verbs

In this section, semantic fusion in simple sentence constructions containing two verbs is discussed in detail to check the operability and rationality of the semantic fusion model in explaining event alignment. Through the application of the event semantic fusion model, how the semantic components in simple sentence constructions containing two verbs are aligned is presented. The alignment clearly reveals that the realization of syntactic overlap is the result of the double semantic profiling of the same syntactic element and explains the correspondence between the syntactic overlap and the semantic overlap.

(i) Patient-agent semantic fusion

**FIGURE 2:**
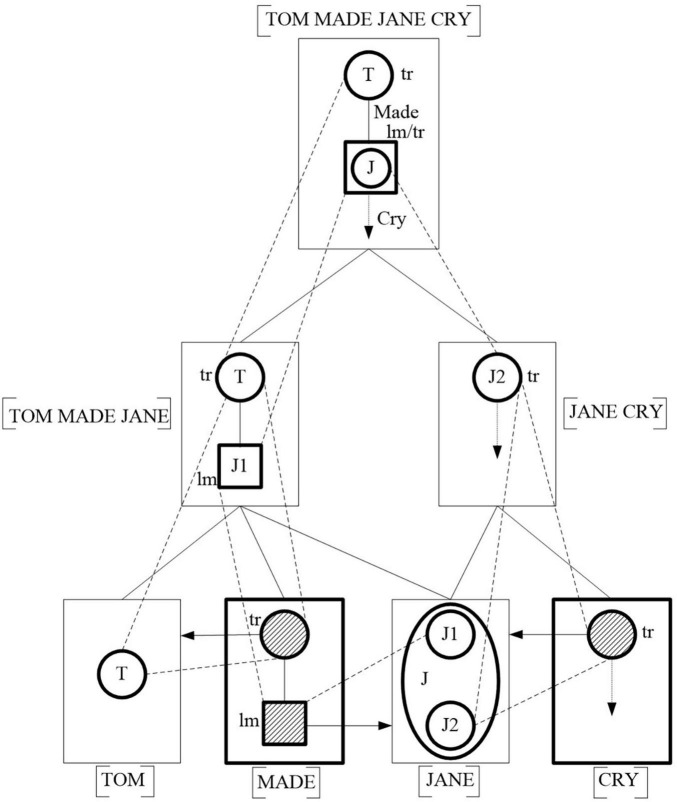
Event alignment in (2) through semantic fusion model.


The realization of the patient-agent semantic fusion is illustrated through the event alignment in (2) in Figure 2.

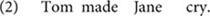


(2) Is patient-agent semantic fusion, with “Jane” being the patient of V_1_ (make) and the agent of V_2_ (cry). V_1_ (make) and V_2_ (cry) are two conceptually dependent components and each of the two verbs semantically profiles a process. The conceptualization of the two verbs needs such components as who performs the action, who is affected in the action, where and when the action happens, etc. “Tom” and “Jane” are two nouns, which are two conceptually autonomous components. Each of the two nouns semantically profiles an entity, making one or more aspects of the entity elevated to a special level of prominence. The semantic profiles of the four elements are shown at the bottom of Figure 2.

**FIGURE 3:**
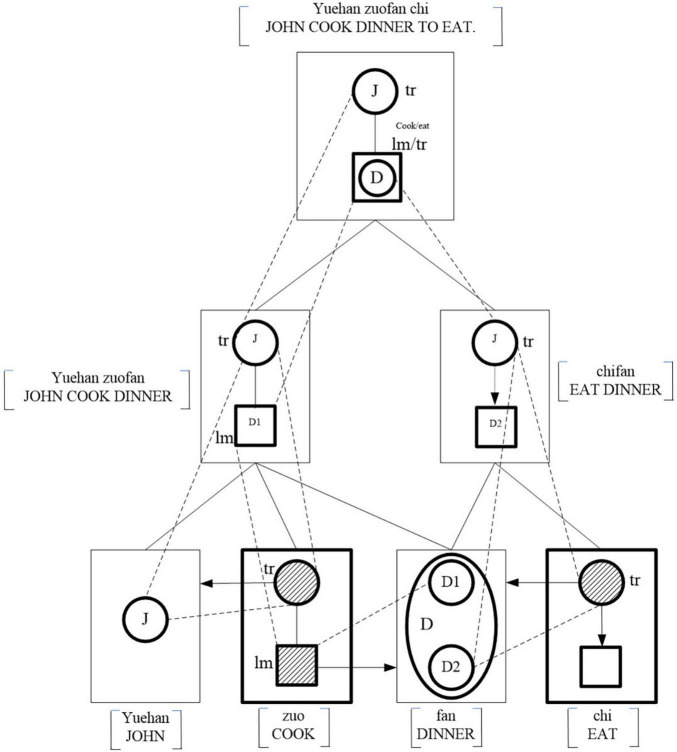
Event alignment in (3) through semantic fusion model.

In (2), the cognitive alignment of the event involves two sub-events: sub-event 1 “Tome made Jane” and sub-event 2 “Jane cry.” Correspondingly, the event alignment includes two parts: one is the combination of sub-event 1, including S (Tom), V_1_ make, and N (Jane), and the other is the combination of sub-event 2, including N (Jane) and V_2_ (cry). In the combination of S (Tom), V_1_ (make), and N (Jane), the dependent element V_1_ (make) profiles a process including a schematic trajector and a schematic landmark as shown at the bottom of Figure 3. S (Tom) profiles an entity capable of performing an action, and N (Jane) profiles an entity that is able to accept action. The semantic profile of S (Tom) elaborates the trajector profiled by V_1_ (make), and “Tom” enters the process and becomes a participant to perform the action expressed by V_1_ (make). The semantic profile of N (Jane) elaborates the landmark profiled by V_1_ (make) and enters the process as a participant who is affected by the action expressed by V_1_ (make). The three elements are aligned into a composite semantic structure [TOM MAKE JANE] that means “Tom did something unpleasant to Jane”. In the combination of N(Jane) and V_2_ (cry), the dependent element V_2_ (cry) profiles a process with a schematic trajector. The semantic profile of N(Jane) elaborates the trajector profiled by V_2_ (cry) and becomes a participant in the process. The two elements are combined into a composite semantic structure [JANE CRY], which means “Jane performs the action of crying.” From the alignments of the two sub-events [TOM MAKE JANE] and [JANE CRY], it is clear that the double semantic profiles of N (Jane) help Jane have the ability to play two roles in the two processes V_1_ (make) and V_2_ (cry) as a participant and that the alignment of a composite semantic structure [TOM MAKE JANE CRY] is realized by the double semantic profiles of the same syntactic element N (Jane), which is regarded as the psychological passage of the two processes.

(ii) Patient-patient semantic fusion

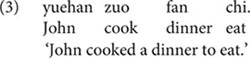


In (3), N (*fan* “dinner”) is the patient of both V_1_ (*zuo* “cook”) and V_2_ (*chi* “eat”). *fan* “dinner” is the patient-patient semantic fusion, with it being the patients of V_1_ (*zuo* “cook”) and V_2_ (*chi* “eat”). In (3), among the four syntactic elements, *Yuehan* “John,” *zuo* “cook,” *fan* “dinner,” and *chi* “eat”; *Yuehan* “John” and *fan* “dinner” are two autonomous elements. *Yuehan* “John” profiles a person capable of performing an action and *fan* “dinner” profiles a thing that can be cooked and eaten. V_1_ (*zuo* “cook”) and V_2_ (*chi* “eat”) are two dependent elements, each profiling a process with a schematic trajector and a schematic landmark. The semantic profiles of the four elements are shown in Figure 3.

The psychological cognition of the event in (3) involves two sub-events “John cooked a dinner” and “Dinner was eaten.” In the alignment of the sub-event “John cooked a dinner,” S (*yuehan* “John”), V_1_ (*zuo* “cook”), and N (*fan* “dinner”), the semantic profiles of S (*yuehan* “John”) and N (*fan* “dinner”) elaborate the schematic trajector and landmark provided by V_1_ (*zuo* “cook”) and the elaboration site into S (*yuehan* “John”) and the landmark into N (*fan* “dinner”). S (*yuehan* “John”) and N (*fan* “dinner”) enter the process profiled by V_1_ (*zuo* “cook”) as participants, and the three elements are aligned into a composite structure [JOHN COOK DINNER], which means “John cooked a dinner.” In the alignment of the sub-event “Dinner was eaten,” the semantic profile of N (*fan* “dinner”) elaborates the schematic landmark provided by V_2_ (*chi* “eat”). Therefore, N (*fan* “dinner”) gets a participant membership and enters the process profiled by V_2_ (*chi* “eat”), and the two elements are aligned into the composite structure [DINNER EATEN], which means “The dinner was eaten.” From the composition of two sub-events, it is discovered that N (*fan* “dinner”) is activated in the two processes and the fusion of two processes into one complete event [JOHN COOK DINNER TO EAT] is realized by the double semantic profiles of N (*fan* “dinner”).

(iii) Other three types of semantic fusion

Through the detailed illustration of patient/agent and patient/patient fusion based on the semantic fusion model, it is discovered that the model works very well in explaining the alignment of events in simple sentence constructions containing two verbs. Through the verification of the event alignment in agent/agent, agent/experiencer, and patient/experiencer semantic fusion types by using the semantic fusion model, the model is found to work in the same way as the event alignment in patient/agent and patient/patient semantic fusion types. So, here the detailed alignment process is not provided with more examples and figures. But one point is very apparent; in agent/agent, agent/experiencer, and patient/experiencer semantic fusion types, N’s two semantic profiles sanction its entry into two processes profiled by V_1_ and V_2_ and the two processes are aligned by N’s simultaneous participation. The simultaneous participation makes N a psychological passage for V_1_ and V_2_ to align into a composite event.

In this section, the realization of semantic fusion is discussed by giving an exact account of the event alignment in Chinese and English simple sentence constructions containing two verbs. From the illustration of the event alignment in simple sentence constructions, it is clear that the double semantic profiles of the same syntactic element offer a convincing explanation for the correspondence between double syntactic identities and the double semantic roles. The alignment process of events in these constructions reveals that the two processes are combined into a composite event through the psychological passage N.

(iv) Discussion of the test results

The 20 constructions in the test are in three different forms: SV_1_NV_2_, SV_1_V_2_N, and SV_1_NAdj. The results of the three tasks in the test are analyzed using R software (R Core Team, 2021). Here, a few sample test constructions are listed in Table 2.

**TABLE 2:** Three sample test constructions.

Forms of the construction	Construction in Chinese/English	Three tasks for each construction
SV_1_NV_2_	*zhangsan qing lisi zuo baogao.* Zhang San invite Li Si make report Zhang San invites Li Si to make a report.	➀ The noun underlined has two semantic roles Yes() No () ➁ construction judgment: SV_1_NV_2_ () SV_1_V_2_N ()
SV_1_V_2_N SV_1_NAdj.	I saw Tom come out of the house. *women da yingle daishou.* we beat succeed opponent *dajia taoyan xiaoliu xuwei.* everyone detest Xiaoliu hypocritical Everyone dislike Xiaoliu because he is hypocritical.	SV_1_NAdj. () ➂ V_1_ and V_2_ are aligned into an event through lisi’ Li Si’ Agree () Basically agree () Disagree () Basically disagree ()

**FIGURE 4:**
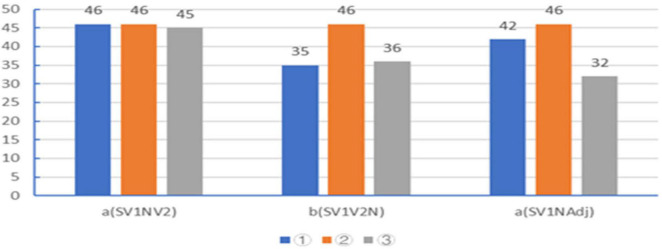
The positive distribution of event semantic fusion in the test.

The positive results of the three tasks are revealed in Figure 4 (the blue, orange, and gray columns in the figure stand for the three tasks of the test, which are numbered as ➀, ➁, and ➂, respectively). About task ➀ in the test, among the 48 participants, on average, 46 participants agree that N possesses two semantic roles in SV_1_NV_2_, 35 participants maintain that N has two semantic roles in SV_1_V_2_N, and 42 participants point out the N’s two semantic roles in AV_1_NAdj. About task ➁, on average, 46 participants can clearly differentiate the three linguistic structures, namely, a (SV_1_NV_2_), b (SV_1_V_2_N), and c (SV_1_NAdj.). About task ➂, on average, 45 participants choose “agree” or “basically agree,” which means they agree that V_1_ and V_2_ are aligned into a complete event through N in SV_1_NV_2_ constructions. Averagely, two participants choose “disagree” that N plays a bond function in the event formation in SV_1_NV_2_ constructions. On average, 36 participants choose “agree” and nine participants choose “disagree” about the bond function of N in the formation in SV_1_V_2_N constructions. About SV_1_NAdj. constructions, the results of the test are as follows: 32 participants, on average, agree or basically agree that N is the bond in combining V_1_ and V_2_ into an event. From the results of the test, it is clear that the syntactic element N is very crucial in the alignment of the event in SV_1_NV_2_ constructions. N becomes the psychological passage in the cognitive alignment of the event in SV_1_NV_2_ constructions. Through the verification of the semantic fusion model in Chinese and English constructions, it is found that the semantic fusion model is effective in the explanation of event cognitive alignment and that semantic fusion has solid psychological reality and is, in essence, a basic cognitive ability for people to perceive and process events in the objective world. The results of the test clearly reveal that semantic roles (semantic role overlap/fusion) have a strong psychological reality. The bond function of the syntactic element N in connecting two actions into an event in language has a strong psychological reality.

## Major Findings

This study, based on the observation and investigation of a large collection of data and the empirical testing of semantic fusion model in Chinese and English simple sentence constructions containing two verbs, establishes a cross-lingual, cognitive model of event alignment by means of semantic fusion and provides a new perspective for the study of event integration in language. Through the analysis of semantic fusion in both Chinese and English simple sentence constructions containing two verbs, it is found that semantic fusion as the event integration and construal is not language-specific and that semantic fusion is the necessary psychological condition for the alignment of semantic components in construction.

## Limitations and Future Concerns

This study has provided a cognitive explanation for event alignment based on the theory of psychology and cognitive linguistics. However, there are some limitations that are insightful for future concerns. First, the data coverage is relatively small. A large-scale corpus from more languages will make a more comprehensive picture of the cognitive mechanism of event alignment in language. Second, the study is an empirical analysis, which needs to be supported by complicated ERP experiments to prove the effectiveness of the semantic fusion model in the event alignment. Third, the role that the conceptualizer plays in the understanding of event alignment is also a future concern.

## Conclusion

This study explores the cognitive alignment of events in Chinese and English simple sentence constructions containing two verbs within the framework of psychology and cognitive linguistics and finds that semantic fusion is rooted in the mental conceptualization of language users and is a common psychological and cognitive behavior. Through the double semantic profiling of an autonomous element, the different attributes of an entity are doubly activated, allowing it to enter different processes as a participant. The double semantic profiles of the same autonomous element make it have the ability to fuse two semantic roles into one participant, establishing a psychological passage by which speakers or conceptualizers process events in constructions.

## Author Contributions

XL has made a direct and intellectual contribution to the work and got it ready for its publication.

## Conflict of Interest

The author declares that the research was conducted in the absence of any commercial or financial relationships that could be construed as a potential conflict of interest.

## Publisher’s Note

All claims expressed in this article are solely those of the authors and do not necessarily represent those of their affiliated organizations, or those of the publisher, the editors and the reviewers. Any product that may be evaluated in this article, or claim that may be made by its manufacturer, is not guaranteed or endorsed by the publisher.

## References

[B1] Allan K. (1986). *Linguistic meaning: Vols. 2.* London: Routledge.

[B2] Blackburn J. M. (1940). A review of gestalt psychology. *J. Ment. Sci.*. 360 1–28. 10.1192/bjp.86.360.1

[B3] Bicknell K. Elman J. L. Hare M. McRae K. Kutas M. (2010). Effects of event knowledge in processing verbal arguments. *J. Memor. Lang.* 63 489–505. 10.1016/j.jml.2010.08.004 21076629 PMC2976562

[B4] Boeckx C. Horstein N. (2004). Movement under control. *Linguist. Inquir.* 2 431–452. 10.1162/0024389041402625

[B5] Bloomfield L. (2002). *Language.* Beijing: Foreign Language Teaching and Research Press.

[B6] Carey S. (2011). Précis of the Origin of Concepts. *Behav. Brain Sci.* 34 113–124. 10.1017/S0140525X10000919 21676291 PMC3489495

[B7] Carlson G. (1998). “Thematic Roles and the Individuation of Events,” in *Events and Grammar, vol 70*, ed. Rothstein S. (Dordrecht: Springer), 35–51. 10.1007/978-94-011-3969-4_3

[B8] Chen J. (2021). “Research and Construction of the Holistic View of Book Binding Design from the Perspective of Gestalt Psychology,” in *Proceedings of 2nd International Conference on Humanities, Arts, and Social Science* (HASS 2021),(Beijing: Boya Century Publishing) 78–84. 10.26914/c.cnkihy.2021.030073

[B9] Chomsky N. (2010). *Lectures on Government and Binding: The Pisa Lectures.* Berlin: De Gruyter Mouton, 10.1515/9783110884166

[B10] Croft W. (1991). *Syntactic Categories and Grammatical Relations: The Cognitive Organization of Information.* Chicago: University of Chicago Press, 10.2307/416423

[B11] Croft W. (2012). *Verbs: aspect and Causal Structure.* Oxford: Oxford University Press, 10.1093/acprof:oso/9780199248582.001.0001

[B12] Davidson D. (1967). *The Logical Form of Action Sentences.* Oxford: Clarendon Press, 10.1093/0199246270.003.0006

[B13] Dowty D. (1986). “Thematic roles and semantics,” in *Proceedings of the twelfth annual meeting of the Berkeley linguistics society*, (Berkeley: Berkeley Linguistics Society).

[B14] Fauconnier G. Turner M. (1996). “Blending as a central process of grammar,” in *Conceptual Structure, Discourse, and Language*, ed. Goldberg A. (Stanford: CSLI), 113-130.

[B15] Feng L. J. Feng L. P. (2018). Restriction of the event structure to the syntactic structure in verb-copying VR construction. *J. Yunnan Norm. Univ.* 3 40–47. 10.16802/j.cnki.ynsddw.2018.03.005

[B16] Fillmore C. J. (1968). “The case for case,” in *Universals in linguistic theory*, eds Bach E. Harms R. T. (New York: Rinehart and Winston), 1–88.

[B17] Givón T. (1990). *Syntax: a Functional-Typological Introduction:Vol. 2.* Amsterdam: John Benjamins, 10.1075/z.17

[B18] Givón T. (2001). *Syntax: An Introduction Volume II.* Amsterdam: John Benjamins Publishing Company.

[B19] Goldberg A. E. (1995). *Constructions: A Construction Grammar Approach to Argument Structure.* Chicago: University of Chicago Press, 10.1016/S0378-2166(97)81937-6

[B20] Gruber J. S. (1965). *Studies in Lexical Relations.* [Ph.D thesis] Boston: MIT Press.

[B21] Huang C. (1982). *Logical Relations in Chinese and the Theory of Grammar.* New York: Garland Publishing, Inc.

[B22] Imbert C. (2012). Path: Ways typology has walked through it. *Lang.Linguist. Compass* 6 236–258. 10.1002/lnc3.329

[B23] Jackendoff R. (1972). *Semantic Interpretation in Generative Grammar.* Cambridge: MIT Press.

[B24] Jackendoff R. (1976). Toward an Explanatory Semantic Representation. *Linguist. Inquir.* 7 89–150. http://www.jstor.org/stable/4177913 10.1016/j.neuropsychologia.2015.10.023 26493748 PMC4783588

[B25] Jackendoff R. S. (1983). *Semantics and Cognition.* Cambridge: MIT Press.

[B26] Jackendoff R. (1990). *Semantic Structures.* Cambridge: MIT Press.

[B27] Jiang K. X. Zhao Y. H. Cui R. Y. (2021). Natural language sentence matching method fusion of high-level and low-level semantic information. *Appl. Res. Comput.* 4:6. 10.19734/j.issn.1001-3695.2021.09.0397

[B28] Kistler M. (2006). *Causation and Laws of Nature.* London: Routledge.

[B29] Koffka F. (1935). *Principles of Gestalt Psychology.* London: Kegan Paul.

[B30] Langacker R. W. (1987). *Foundations of Cognitive Grammar: Theoretical Prerequisites. Vol. 1.* Stanford: Stanford University Press.

[B31] Langacker R. W. (2012). Access, Activation, and Overlap: Focusing on the Differential. *J.Foreig. Lang.* 1 1–24.

[B32] Levin B. (1993). *English Verb Classes and Alternations.* Chicago: The University of Chicago Press.

[B33] Li F. Y. (2019). Evolutionary order of macro-events in Mandarin. *Rev.Cogn. Ling.* 17 155–186. 10.1075/rcl.00030.li 33486653

[B34] Li F. Y. (2020). Macro-event Hypothesis and its empirical studies in Mandarin. *Foreign Lang. Teach.Res.* 3 349–360. 10.19923/j.cnki.fltr.2020.03.003

[B35] Li X. L. (2015). *A Cognitive Grammar Approach to Semantic Overlapping and Discourse Funciton in Jianyu Construction in Chinese.* Beijing: Science Press.

[B36] Li X. L. (2019). On the cognitive mechanism of syntactic alignment through semantic overlap-ping in Chinese and English. *Stud. Foreign Lang.* 3 11–18.

[B37] Liu D. Q. (2017). Split between Verb-complement Constructions and Serial Verb Constructions in Chinese Syntactic Inventory. *Lang. Teach. Linguist. Stud.* 2 1–16.

[B38] Lv S. X. (1979). *Grammatical analysis of Chinese.* Beijing: The Commercial Press.

[B39] Metusalema R. Kutas M. Urbacha T. P. Hare M. McRae K. Elmana J. L. (2012). Generalized event knowledge activation during online sentence comprehension. *J. Mem. Lang.* 4, 545–567. 10.1016/j.jml.2012.01.001 22711976 PMC3375826

[B40] Michotte A. E. (1946). *The Perception of Causality.* New York: Basic Books.

[B41] Niu B. Y. (2008). A cognitive analytical model of Autonomy/Dependence Alignment. *Foreign Lang. Their Teach.* 1 1–5.

[B42] Parsons T. (1990). *Events in Semantics of English: A Study in Subatomic Semantics.* Cambridge: MIT Press.

[B43] Pinker S. (1989). *Learnability and Cognition: The Acquisition of Argument Structure.* Cambridge: The MIT Press.

[B44] R Core Team (2021). *R: A Language and Environment for Statistical Computing.* Vienna: R Foundation for Statistical Computing.

[B45] Rappaport H. M. (2008). “Lexicalized meaning and the internal temporal structure of events,” in *Theoretical and Crosslinguistic Approaches to the Semantics of Aspect*, ed Rothstein S. (Rothstein Amsterdam: John Benjamins), 13–42. ed. S. 10.1075/la.110.03hov

[B46] Rissman L. Majid A. (2019). Thematic roles: Core knowledge or linguistic construct? *Psychon. Bull. Rev.* 26 1850–1869. 10.3758/s13423-019-01634-5 31290008 PMC6863944

[B47] Sato M Niikuni K Schafer A. J. Koizumi M. (2020). Agentive versus non-agentive motions immediately influence event apprehension and description: an eye-tracking study in a VOS language. *J. East Asian Linguist.* 4 211–236. 10.1007/s10831-020-09205-9

[B48] Shipley T. Zacks J. (2008). *Understanding Events.* Oxford: Oxford University Press.

[B49] Strickland B. (2017). Language Reflects “Core” Cognition: A New Theory About the Origin of Cross-Linguistic Regularities. *Cognitive Sci.* 1 70–101. 10.1111/cogs.12332 26923431

[B50] Talmy L. (1985). “Lexicalization patterns: Semantic structure in lexical forms,” in *Language Typology and Syntactic Description. Vol. III: Grammatical Categories and the Lexicon*, ed. Shopen T. (Cambridge: CUP).

[B51] Talmy L. (1991). “Path to realization: A typology of event conflation,” in *Proceedings of the17th Annual Meeting of the Berkeley Linguistics Society*, (Berkeley: Berkeley Linguistics Society), 480-519.

[B52] Talmy L. (2000a). *Toward a Cognitive Semantics. Vol.1.* Cambridge: the MIT Press.

[B53] Talmy L. (2000b). *Toward a Cognitive Semantics. Volume II: Typology and Process in Concept Structuring.* Cambridge: the MIT Press.

[B54] Van V. R. D. (2005). *Exploring the Syntax-Semantics Interface.* Cambridge: Cambridge University Press.

[B55] Vendler Z. (1957). Verbs and times. *Philos. Rev.* 66 143–160. 10.2307/2182371

[B56] Viswanatha N. Zlatev J Duggirala V. Weijer J, V, D. (2018). Holistic s patial semantics and post-Talmian motion event typology: A case study of Thai and Telugu. *Cognitive Semiotics* 2 1–27.

[B57] Wen X. Yin H. (2018). Event Structure Analysis of the Constructionalization of De Emotion-caused Constructions. *J. Northwest Norm. Univ.* 4 50–55. 10.16783/j.cnki.nwnus2018.04.006

[B58] Wolff P. (2003). Direct causation in the linguistic coding and individuation of causal events. *Cognition* 88 1–48. 10.1016/S0010-0277(03)00004-0 12711152

[B59] Wu J. C. Liang B. S. (1992). *Sentence Structure of Modern Chinese and Its Analysis.* Beijing: Language and Culture Press.

[B60] Wu Y. J. Thierry G. (2011). Event-related brain potential investigation of preparation for speech production in late bilinguals. *Front. Psychol.* 2:114. 10.3389/fpsyg.2011.00114 21687468 PMC3108551

[B61] Wu Y. J. Thierry G. (2017). Brain potentials predict language selection before speech onset in bilinguals. *Brain Lang.* 171 23–30. 10.1016/j.bandl.2017.04.002 28445784

[B62] Xing X. (2004). *Jianyu Construction in Modern Chinese.* Beijing: Communication University of China Press.

[B63] Xing F. Y. (1986). *Modern Chinese.* Beijing: Higher Education Press.

[B64] Zhang H. L. Pan H. H. (2019). Inflectional rhyme change and the grammaticalization of event structure. *Stud.Chinese Lang.* 1 74–88.

[B65] Zhang Y. F. (1999). Pragmatic studies of “having sentence”. *J. Henan Univ. Soc. Sci.* 3, 19–23.

[B66] Zhang Y. J. (2001). *Grammatics of Inscriptions on Tortoise Shells of the Shang Dynasty.* Shanghai: Academic Press.

